# Cytotoxic T Cells Drive Outcome in Inflammatory Dilated Cardiomyopathy

**DOI:** 10.1161/CIRCRESAHA.124.325183

**Published:** 2024-10-21

**Authors:** Maurits A. Sikking, Daniel Harding, Michiel T.H.M. Henkens, Sophie L.V.M. Stroeks, Max F.G.H.M. Venner, Bastien Nihant, Rick E.W. van Leeuwen, Silvia Fanti, Xiaofei Li, Pieter van Paassen, Christian Knackstedt, Hans-Peter Brunner-la Rocca, Vanessa P.M. van Empel, Job A.J. Verdonschot, Federica M. Marelli-Berg, Stephane R.B. Heymans

**Affiliations:** Department of Cardiology, Cardiovascular Research Institute Maastricht (M.A.S., M.T.H.M.H., S.L.V.M.S., M.F.G.H.M.V., B.N., R.E.W.v.L., C.K., H.-P.B.-L.R., V.P.M.v.E., J.A.J.V., S.R.B.H.), Maastricht University Medical Center, the Netherlands.; Department of Pathology (M.T.H.M.H., X.L.), Maastricht University Medical Center, the Netherlands.; Division Clinical and Experimental Immunology, Department of Internal Medicine (P.v.P.), Maastricht University Medical Center, the Netherlands.; Department of Clinical Genetics (S.L.V.M.S., J.A.J.V.), Maastricht University Medical Center, the Netherlands.; William Harvey Research Institute, Barts and The London School of Medicine and Dentistry (D.H., S.F., F.M.M.-B.), Queen Mary University of London, United Kingdom.; Centre for Inflammation and Therapeutic Innovation (F.M.M.-B.), Queen Mary University of London, United Kingdom.; Barts Heart Centre, Barts Health NHS Trust, St Bartholomew’s Hospital, London, United Kingdom (D.H., F.M.M.-B.).; Netherlands Heart Institute, Utrecht, the Netherlands (M.T.H.M.H.).; Maastricht UMC+ European Reference Network for Rare, Low Prevalence and Complex Diseases of the Heart (ERN GUARD-Heart) (S.L.V.M.S., J.A.J.V., S.R.B.H.).; Department of Cardiovascular Sciences, Centre for Molecular and Vascular Biology, Herestraat 49, University of Leuven, Belgium (S.L.V.M.S., S.R.B.H.).

**Keywords:** allergy and immunology, cardiomyopathy, dilated, heart failure, leukocytes, mononuclear, myocarditis

Dilated cardiomyopathy (DCM) is often driven by myocarditis, a condition known as inflammatory DCM (infl-DCM). Infl-DCM classifies as an immune-mediated disease according to the Rose-Witebsky criteria. To date, most evidence points to IL (interleukin)-17 expressing helper T cells (Th17 cells) as drivers of the progression from acute myocarditis to infl-DCM (eg, the study by Myers et al^1^). Research on the role of cytotoxic T cells (Tc cells) is lacking.

To investigate the adaptive T cell–mediated immune responses in infl-DCM, comprehensive immunophenotyping was performed in peripheral blood mononuclear cells from 125 patients with nonischemic, nonvalvular DCM undergoing endomyocardial biopsy (EMB; median time since DCM diagnosis, 9 [4–26] months), excluding patients with systemic immune diseases (n=6). Flow cytometry was performed on T cells and innate immune cells. Plasma levels of IL-6 and TGF (tumor growth factor)-β, which drive Th17-differentiation (eg, the study by Myers et al^1^), were measured by ELISA. Patients were allocated to 3 groups: infl-DCM (≥7 T cells/mm^2^ or ≥14 leukocytes/mm^2^ in EMB; n=20), non-infl-DCM (<3 T cells/mm^2^ and <5 leukocytes/mm^2^; n=37), and patients not fulfilling these criteria (n=68). A combined outcome of all-cause mortality, heart failure hospitalization, and life-threatening arrhythmia was assessed over a 2-year period (median duration, 8.5 [4.3–25.7] months). Findings were correlated with RNA sequencing of EMBs in a cohort of 41 patients. Normality was assessed by using Q-Q plots and histograms. All analyses were performed with R (version 4.0.4) using the DESeq2 and biomaRt packages. This study adhered to the Helsinki Declaration and obtained ethics approval and patient consent.

Clinical characteristics were similar between infl-DCM and non-infl-DCM (Figure [A]). Helper T cells more often expressed IL-17A in infl-DCM compared with non-infl-DCM (17.8±5.1% versus 13.5±5.4%; *P*=0.04). Interestingly, the cardiac transcript of *IL-17RC*, part of the IL-17A receptor complex, was increased in infl-DCM compared with non-infl-DCM (Figure [E]). However, the expression of IL-6 or TGF-β did not significantly differ, when measured in plasma (protein), circulating monocytes and dendritic cells (RNA), or EMBs (RNA; Figure [E]).

**Figure.**
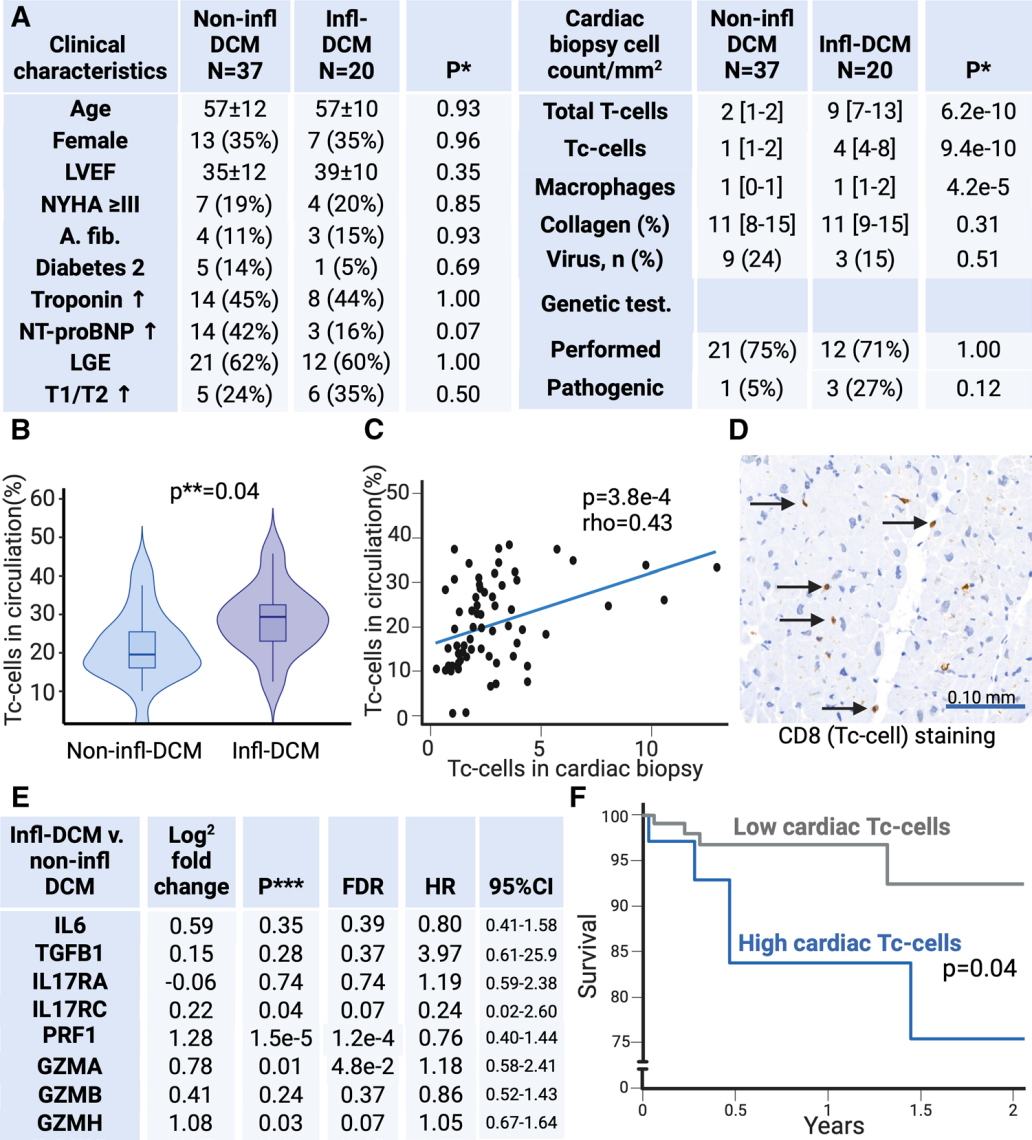
**Circulating and cardiac cytotoxic T cells (Tc cells) drive poor outcomes in inflammatory dilated cardiomyopathy. A**, Clinical characteristics comparison between non-inflammatory DCM (non-infl-DCM) and inflammatory DCM (infl-DCM) shows no between-group differences. However, cardiac T cells and Tc cells and macrophages are increased in infl-DCM compared with non-infl-DCM (*P*=6.2×10^−10^, *P*=9.4×10^−10^, and *P*=4.2×10^−5^, respectively). Genetic mutations were found in *TTN*, *FLNC*, and *MYBPC3* in infl-DCM and *MYH7* in non-infl-DCM. **B**, The proportion of circulating Tc cells in infl-DCM was increased compared with non-infl-DCM (*P*=0.04). **C**, In the total study cohort (n=125), the proportion of circulating Tc cells correlated with the density of Tc cells in endomyocardial biopsy (EMB; Spearmanρ=0.43; *P*=3.8×10^−4^). **D**, An example of Tc cell staining of an EMB of infl-DCM. **E**, Comparison of cardiac gene expression by transcriptomics of EMBs of non-infl-DCM and infl-DCM patients shows higher expression of *PRF1* (perforin-1), *GZMA* (granzyme-A), *GZMH* (granzyme-H), and *IL17RC* (interleukin-17 receptor-C), after correcting for multiple testing (false discovery rate [FDR] <0.1 as per Benjamini-Hochberg procedure). Expression of these genes was not associated with prognosis. **F**, In the total study cohort (n=125), high cardiac Tc cells (≥3 Tc cells/mm^2^ corresponding with the third quartile of cardiac Tc cells) correlated with a worse prognosis compared with low cardiac Tc cells (<3 Tc cells/mm^2^) as tested by log-rank test (*P*=0.04). *Fisher exact or Mann-Whitney *U* test; **Mann-Whitney *U* test; and ***2-sample *t* tests. a. fib indicates atrial fibrillation; CD, cluster of differentiation; GZMB, granzyme-B; IL17RA, interleukin-17 receptor-A; IL-6, interleukin-6; LGE, late-gadolinium enhancement; LVEF, left ventricle ejection fraction; NT-proBNP, N-terminal pro-B-type natriuretic peptide; NYHA, New York Heart Association dyspnea classification; T1/T2, T1- or T2-weighted imaging; and TGFB1, tumor growth factor-β1.

In infl-DCM, a Tc cell–driven immune reaction stood out as a dominant feature of the ongoing immune response (Figure [B–F]). Tc cells were increased in EMBs (Figure [A, C, and D]) and in the circulation (Figure [B]) of patients with infl-DCM compared with non-infl-DCM. Crucially, a higher proportion of Tc cells expressed granzyme-B in infl-DCM (50.7% [40.8–73.0] versus 33.1% [16.6–57.8] in non-infl-DCM; *P*=0.01), indicating enhanced cytolytic activity. These Tc cells also shifted towards an exhausted phenotype and displayed markers associated with CD45RA re-expression (TEMRA [cytotoxic T-cells with markers associated with CD45RA re-expression] cells; CD8+/CCR7-/CD45RO-/CD57+/KLRG-1+/PD-1+; 0.6% [0.4–1.7] versus 0.3% [0.1–0.6]; *P*=0.02). The frequency of circulating Tc cells directly correlated with their density in EMBs (Figure [C]), suggesting migration of these cells to the myocardium. This conclusion was reinforced by increased Tc-cell gene transcription in infl-DCM compared with non-infl-DCM EMBs (Figure [E]). Cardiac T cell infiltration predicted poor patient outcomes (Figure [F]), highlighting their role in infl-DCM progression.

Our study reveals Tc cell–mediated immunity as a dominant pathway in infl-DCM, possibly explained by an upregulated Th17 response. Heart-infiltrating Tc cells induce cardiomyocyte apoptosis and adverse remodeling via granzyme release.^2^ Th17 cells promote T cell exhaustion and enhance cellular cytotoxicity.^3^ Thus, Th17-mediated immunity may enhance T cell activity and subsequent adverse cardiac remodeling. Based on these findings, downregulating T cell activity by targeted therapy may hold promise to halt infl-DCM progression.

This study could not confirm the upregulation of the IL-6/TGF-β pathway as the main driver behind the observed increase in IL-17A response. While IL-6 and TGF-β are important for the differentiation of Th17 cells, IL-23 may sustain preexisting Th17 responses in the absence of IL-6 and TGF-β.^1^ Finally, the drivers of the T cell response remain yet to be elucidated. A viral cause of the increased T cell response was not found.

The strength of this study includes the performance of comprehensive immune phenotyping and follow-up of patients with biopsy-confirmed infl-DCM and non-infl-DCM. Limitations include the relatively short follow-up period and the lack of a validation cohort. Despite this, our study reveals a key role for Tc cell–driven immune response as a key pathogenic feature in infl-DCM. Therefore, Tc cell–targeting immunotherapies may, therefore, be considered in infl-DCM.

## ARTICLE INFORMATION

### Acknowledgments

Figure was created by M.A.S. via BioRender.com.

### Sources of Funding

The authors acknowledge the Double Dose Program 2020-B005, CVONArena-PRIME-2017-18, HORIZON-EIC-2022-PATHFINDERCHALLENGES, ZonMW-Metacor, and British Heart Foundation grants FS/CRTF/20/24058, RG/20/8/34995, CH/15/2/32064, and AA/18/5/34222.

### Disclosures

S.R.B. Heymans receives fees for scientific advice for AstraZeneca, Ribocure, and CSL Behring and receives research support from AstraZeneca and CSL Behring. The other authors report no conflicts.

### Data Availability

Data are available in the Major Resources Table in the Supplemental Material.

## Supplementary Material


