# Supplementary material for: Cytotoxic T Cells Drive Outcome in Inflammatory Dilated Cardiomyopathy
Source: Circ Res. 2024 Oct 21;135(12):1193–5. doi: 10.1161/CIRCRESAHA.124.325183 (PMC11620291; doi:10.1161/CIRCRESAHA.124.325183)
Supplement: Supplementary file 1 [file res-135-1193-s001.pdf]

Major Resources Table

In order to allow validation and replication of experiments, all essential research materials listed in the Methods should be included in the Major Resources Table below. Authors are encouraged to use public repositories for protocols, data, code, and other materials and provide persistent identifiers and/or links to repositories when available. Authors may add or delete rows as needed.

Antibodies

Target antigen	Vendor or Source	Catalog #	Working concentration
c-Met	Thermofisher	11-8858-42	As described by manufacturer's instructions
IL-10	Biolegend	501418	As described by manufacturer's instructions
CD45RO	Biolegend	304224	As described by manufacturer's instructions
Aqua	Biolegend	353232	As described by manufacturer's instructions
IFN-g	Biolegend	506542	As described by manufacturer's instructions
CD8	Biolegend	344730	As described by manufacturer's instructions
IL-17A	Biolegend	512338	As described by manufacturer's instructions
IL-4	Biolegend	500812	As described by manufacturer's instructions
Perforin	Biolegend	353324	As described by manufacturer's instructions
CD3	Biolegend	300318	As described by manufacturer's instructions
IL-22	Biolegend	366716	As described by manufacturer's instructions
CD4	Biolegend	317412	As described by manufacturer's instructions
GrzB	Biolegend	372214	As described by manufacturer's instructions
TGF (LAP)	Biolegend	300010	As described by manufacturer's instructions
CD56	Biolegend	304626	As described by manufacturer's instructions
CX3CR1	Biolegend	341620	As described by manufacturer's instructions
Aqua			As described by manufacturer's instructions
HLA-DR	Thermofisher	63-9956-42	As described by manufacturer's instructions
CD3	Biolegend	317324	As described by manufacturer's instructions
CD16	Thermofisher	78-0168-42	As described by manufacturer's instructions
IL-1β	Biolegend	511707	As described by manufacturer's instructions
CD88	Biolegend	344314	As described by manufacturer's instructions
Cd11c	Biolegend	337218	As described by manufacturer's instructions
IL-12	Biolegend	501807	As described by manufacturer's instructions
TNF-alpha	Biolegend	502946	As described by manufacturer's instructions
CD14	Thermofisher	15-0149-42	As described by manufacturer's instructions
IL-6	Biolegend	501120	As described by manufacturer's instructions
c-Met	Thermofisher	11-8858-42	As described by manufacturer's instructions
PD-1 (CD279)	Biolegend	621614	As described by manufacturer's instructions
CD45RO	Biolegend	304224	As described by manufacturer's instructions
Aqua			As described by manufacturer's instructions

DOI [to be added]

KLRG1	Thermofisher	63-9488-42	As described by manufacturer's instructions
CD27	Biolegend	302828	As described by manufacturer's instructions
TIM-3	Biolegend	345032	As described by manufacturer's instructions
LAG-3	Biolegend	369212	As described by manufacturer's instructions
CD8	Biolegend	344724	As described by manufacturer's instructions
CD3	Biolegend	300318	As described by manufacturer's instructions
CCR7	Biolegend	353204	As described by manufacturer's instructions
CD57	Biolegend	359620	As described by manufacturer's instructions
CD4	Biolegend	317412	As described by manufacturer's instructions
CTLA-4	Thermofisher	25-1529-42	As described by manufacturer's instructions

Data & Code Availability

Description	Source / Repository	Persistent ID / URL
RNA sequencing of endomyocardial biopsies of n=41 patients with dilated cardiomyopathy	GEO Gene Expression Omnibus; GEO accession number GSE278521	https://www.ncbi.nlm.nih.gov/geo/query/acc.cgi?acc=GSE278521

DOI [to be added]

STROBE Statement—checklist of items that should be included in reports of observational studies

	Item No.	Recommendation	Page No.
**Title and abstract**	1	(*a*) Indicate the study's design with a commonly used term in the title or the abstract	1
		(*b*) Provide in the abstract an informative and balanced summary of what was done and what was found	NA
**Introduction**			
Background/rationale	2	Explain the scientific background and rationale for the investigation being reported	2
Objectives	3	State specific objectives, including any prespecified hypotheses	2
**Methods**			
Study design	4	Present key elements of study design early in the paper	2
Setting	5	Describe the setting, locations, and relevant dates, including periods of recruitment, exposure, follow-up, and data collection	2
Participants	6	(*a*) *Cohort study*—Give the eligibility criteria, and the sources and methods of selection of participants. Describe methods of follow-up<br>*Case-control study*—Give the eligibility criteria, and the sources and methods of case ascertainment and control selection. Give the rationale for the choice of cases and controls<br>*Cross-sectional study*—Give the eligibility criteria, and the sources and methods of selection of participants	2
		(*b*) *Cohort study*—For matched studies, give matching criteria and number of exposed and unexposed<br>*Case-control study*—For matched studies, give matching criteria and the number of controls per case	NA
Variables	7	Clearly define all outcomes, exposures, predictors, potential confounders, and effect modifiers. Give diagnostic criteria, if applicable	2
Data sources/ measurement	8*	For each variable of interest, give sources of data and details of methods of assessment (measurement). Describe comparability of assessment methods if there is more than one group	2
Bias	9	Describe any efforts to address potential sources of bias	NA
Study size	10	Explain how the study size was arrived at	NA

Continued on next page

Quantitative variables	11	Explain how quantitative variables were handled in the analyses. If applicable, describe which groupings were chosen and why	2
Statistical methods	12	(*a*) Describe all statistical methods, including those used to control for confounding	2
		(*b*) Describe any methods used to examine subgroups and interactions	2
		(*c*) Explain how missing data were addressed	NA
		(*d*) *Cohort study*—If applicable, explain how loss to follow-up was addressed<br>*Case-control study*—If applicable, explain how matching of cases and controls was addressed<br>*Cross-sectional study*—If applicable, describe analytical methods taking account of sampling strategy	NA
		(*e*) Describe any sensitivity analyses	NA
**Results**			
Participants	13*	(a) Report numbers of individuals at each stage of study—eg numbers potentially eligible, examined for eligibility, confirmed eligible, included in the study, completing follow-up, and analysed	NA
		(b) Give reasons for non-participation at each stage	NA
		(c) Consider use of a flow diagram	NA
Descriptive data	14*	(a) Give characteristics of study participants (eg demographic, clinical, social) and information on exposures and potential confounders	7
		(b) Indicate number of participants with missing data for each variable of interest	NA
		(c) *Cohort study*—Summarise follow-up time (eg, average and total amount)	2
Outcome data	15*	*Cohort study*—Report numbers of outcome events or summary measures over time	7
		Case-control study—Report numbers in each exposure category, or summary measures of exposure	
		Cross-sectional study—Report numbers of outcome events or summary measures	
Main results	16	(*a*) Give unadjusted estimates and, if applicable, confounder-adjusted estimates and their precision (eg, 95% confidence interval). Make clear which confounders were adjusted for and why they were included	3
		(*b*) Report category boundaries when continuous variables were categorized	3
		(*c*) If relevant, consider translating estimates of relative risk into absolute risk for a meaningful time period	NA

Continued on next page

DOI [to be added]

Other analyses	17	Report other analyses done—eg analyses of subgroups and interactions, and sensitivity analyses	NA
**Discussion**			
Key results	18	Summarise key results with reference to study objectives	4
Limitations	19	Discuss limitations of the study, taking into account sources of potential bias or imprecision. Discuss both direction and magnitude of any potential bias	4
Interpretation	20	Give a cautious overall interpretation of results considering objectives, limitations, multiplicity of analyses, results from similar studies, and other relevant evidence	4
Generalisability	21	Discuss the generalisability (external validity) of the study results	4
**Other information**			
Funding	22	Give the source of funding and the role of the funders for the present study and, if applicable, for the original study on which the present article is based	5

*Give information separately for cases and controls in case-control studies and, if applicable, for exposed and unexposed groups in cohort and cross-sectional studies.

Note: An Explanation and Elaboration article discusses each checklist item and gives methodological background and published examples of transparent reporting. The STROBE checklist is best used in conjunction with this article (freely available on the Web sites of PLoS Medicine at http://www.plosmedicine.org/, Annals of Internal Medicine at http://www.annals.org/, and Epidemiology at http://www.epidem.com/). Information on the STROBE Initiative is available at www.strobe-statement.org.
